# Second victim phenomenon: impact on healthcare professionals, organizational responsibility and support strategies

**DOI:** 10.15649/cuidarte.5072

**Published:** 2025-05-01

**Authors:** Maristela Santini Martins, Helena de Rezende, Ellen Regina Sevilla Quadrado, Andresa Gomes de Paula, Hércules de Oliveira Carmo, Vagner Ferreira do Nascimento

**Affiliations:** 1 Escola de Enfermagem da Universidade de São Paulo (EEUSP), PPGEn, GPQUALIS. São Paulo, Brazil. E-mail: maristelasanti@usp.br Escola de Enfermagem da Universidade de São Paulo São Paulo Brazil maristelasanti@usp.br; 2 Bournemouth University. GPQUALIS. Bournemouth, Reino Unido. E-mail: hderezende@bournemouth.ac.uk Bournemouth University Bournemouth Reino Unido hderezende@bournemouth.ac.uk; 3 Juravinski Hospital, Hamilton Health Sciences. GPQUALIS. Hamilton, Ontario, Canadá. E-mail: sevillaqua@hhsc.ca Juravinski Hospital, Hamilton Health Sciences. GPQUALIS Ontario Canadá sevillaqua@hhsc.ca; 4 Escola de Enfermagem da Universidade de São Paulo (EEUSP), PPGEn, GPQUALIS. São Paulo, Brazil. E-mail: andresa.paula@usp.br Escola de Enfermagem da Universidade de São Paulo (EEUSP) São Paulo Brazil andresa.paula@usp.br; 5 Escola de Enfermagem da Universidade de São Paulo (EEUSP), PPGEn, GPQUALIS. São Paulo, Brazil. E-mail: hercules.enf@usp.br Escola de Enfermagem da Universidade de São Paulo (EEUSP) São Paulo Brazil hercules.enf@usp.br; 6 Universidade do Estado de Mato Grosso (UNEMAT), Faculdade Indígena Intercultural. Barra do Bugres, Mato Grosso, Brazil. E-mail: vagnernascimento@unemat.br Universidade do Estado de Mato Grosso (UNEMAT) Mato Grosso Brazil vagnernascimento@unemat.br

Unsafe practices and incidents that result in negative patient outcomes can lead to potential victims. While patients are the primary and most apparent victims, healthcare workers also suffer from their mistakes, in that they experience trauma following the event^1^ and are deemed the second victims^2^. The term "second victim" (SV) was first described by Wu (2000), who proposed that physicians who make mistakes also need help. Later, Scott expanded the concept, defining SVs as professionals involved in a health error^3^. More recently, an international consensus proposed that an SV can be any healthcare worker—whether directly or indirectly involved in an adverse event (AE), unintentional error, or patient-related injury—who is also negatively impacted by the experience of becoming a victim^4^.

The occurrence of errors triggers stressors in the professionals involved, including psychological, cognitive, and/or physical reactions^5^. Prevalent manifestations include memory impairment, anxiety, self-directed anger, remorse, worry, fear of making a mistake again, sleep disturbances, and embarrassment in front of coworkers. Physical symptoms include fatigue, tachycardia, hypertension, tachypnea, and muscle tensionr^6^,^7^. 

In a study involving 31 SVs, six stages were mapped. Initially, in the stage of **responding to the incident and the onset of chaos**, SVs experience emotional and cognitive overload as they grapple with disorganized thoughts, self-reflection, and attempts to understand what happened, all while managing a patient in crisis. Generally, they seek emergency support with the aim of stabilizing the first victim. In the stage of **intrusive reflections**, persistent thoughts of fear and guilt prevail, prompting a re-evaluation of the event and an ongoing search for explanations behind the error. In an attempt to **restore their sense of personal integrity**, SVs turn to trusted individuals, such as colleagues or family members, for support while also confronting self-criticism and concerns about their professional reputation^3^.

Subsequent stages involve more complex institutional and emotional challenges. In the **enduring inquisition stage**, anxieties about legal and disciplinary repercussions arise alongside physical and psychological symptoms, prompting SVs to **the stage of obtaining emotional first aid**. Psychological and legal support becomes critical, driving a need for safe spaces to express distress. In the **moving on—dropping out, surviving or thriving** stage, SVs may follow one of three paths: dropping out, surviving, or thriving, depending on the resilience and support received. This stage determines the event’s long-term impact, potentially leading to career withdrawal, staying but with emotional harm or strengths, and commitment to patient safety initiatives^3^. Not all SVs experience these stages sequentially; some may stall in progression.

The SV phenomenon affects healthcare teams worldwide. In the United Kingdom, 76% of professionals involved in near misses or adverse events reported experiencing emotional impacts^8^. In Central Europe, the phenomenon is also relevant. In Germany^9^, 59% of physicians reported feeling like SV following an AE, while in Austria, 43% reported this experience at least once after incidents of this nature^10^. In Belgium, general practitioners reported high levels of hypervigilance, guilt, stress, and shame. In Spain^11^, 70% of nurses and physicians reported having experienced the SV phenomenon either directly or indirectly, while in Italy, 41% presented psychological and physical symptoms, as well as an intention to leave their job following the event^12^.

Approximately 50% of healthcare professionals in Canada have reported being affected by SV experience at some point in their careers^13^,^14^. In the United States, an estimated 53% of pharmacists and 15% of pharmacy technicians identified themselves as SVs. Among pharmacists, 60% reported that it took between one week and one year to overcome the AE, while 20% reported needing more than a year or never recovering from the post-traumatic event^15^. In another study conducted in the United States with professionals who did not provide direct patient care, 26.7% of them reported having experienced the SV phenomenon throughout their careers, and 13.3% had experienced it within the past year^16^.

In Argentina, most professionals who experienced the SV phenomenon prioritized communicating the AE to their team, patients, and family members, highlighting positive aspects. However, some reported a lack of understanding and acceptance from their supervisors^17^. In Chile, 90.2% of nurses working in Intensive Care Units had been involved in an AE. Among them, 65.6% reported the incident to their supervisor, 66% expressed feelings of guilt about what happened, and 53% reported being aware of institutional support^18^.

A study involving Brazilian nurses revealed the group's difficulty in reporting AEs in the institution where they work due to fear of judgment and punishment, even exhibiting signs and symptoms of emotional distress^19^. In the same country, 54.3% of newly graduated nurses involved in AEs were unfamiliar with the term "second victim." Negative feelings (94.6%) and insecurity (70.3%) were prevalent. While the majority received support (59.5%), not all was provided through formal or institutional channels^20^, a situation consistent with findings from another national study^21^.

The training and preparation of healthcare teams to understand the SV phenomenon, along with the support offered to these professionals, are just as important as reporting errors. However, organizational culture and how leaders handle such situations have a direct impact on the reporting of AEs. These data highlight the urgent need for institutional policies aimed at mitigating this phenomenon's negative effects, which represents a critical challenge for health systems worldwide. Therefore, support strategies for healthcare professionals who experience the SV phenomenon should aim to provide emotional and psychological support, enabling their recovery and return to work.

Formal support programs and services have been developed by hospitals and educational organizations. These programs are similar because they address the SV, offering support across three levels of care. The first level involves initial contact by colleagues or coworkers as soon as possible after the occurrence of an AE. The second level consists of the support provided by professionals trained to assess whether signs of distress persist in the SV. However, if emotional distress persists, these professionals should be referred to specialized care, which may include psychologists and/or legal counselors. Examples of these programs include Peer Support from the Center for Professionalism and Peer Support (CPPS)^22^, Resilience in Stressful Events (RISE)^23^, and Medically Induced Trauma Support Services (MITSS)^24^.

Another support strategy refers to implementing guidelines and tools that provide recommendations to strengthen the culture of safety, develop institutional policies, and offer support to patients, healthcare professionals, and institutions following an AE. This strategy may take the form of guidelines, scripts, checklists, and algorithms^25^, among others. One example is the guideline developed by the Agency for Healthcare Research and Quality (AHRQ)^26^, used to guide managers and professionals in implementing, monitoring, and improving the Care for the Caregiver program. As a model tool, there is the Toolkit for Building a Clinician and Staff Support Program, available from MITSS^27^, which helps develop a culture of safety, train peer supporters, and communicate with SV. Another electronic tool is BACRA (in Spanish Basado en Análisis Causa-Raíz meaning "based on root-cause analysis")^25^, designed to help managers monitor healthcare-related risks of professionals affected by AEs.

The literature also describes other forms of interventions and actions to support SVs, which may be informal or formal. Informal experiences include sharing experiences with colleagues, spouses, family members, friends, or other trusted individuals^23^,^28^,^29^. Formal approaches involve structured dialogues with managers, mental health specialists, or experienced peers trained in this role^28^,^30^,^31^. In addition, problem- and emotion-focused coping strategies^32^,^33^, reflective writing^30^,^34^, temporary leave from work^35^,^36^, learning from mistakes^37^, and receiving positive feedback^38^,^39^ are highlighted. SVs recognize employee assistance programs^14^, institutional policies, and guidelines aimed at protecting the patient-professional relationship as an organizational support strategy^40^,^41^ and resort to a Second Victim Support Unit (USVIC)^42^, an online platform developed to strengthen communication, explain the phenomenon, and provide support for patient safety^39^,^43^.

Leadership support for SV is also critical. Leaders with more collaborative and decentralized profiles, who move away from authoritarian and hierarchical models, foster greater trust among staff, encouraging the reporting of AEs and promoting continuous improvement in care quality and patient safety^44^,^45^. Moreover, how leaders support SVs is crucial for shaping the outcome of this condition. In Finland, nurse managers recommend peer support for SVs, independent of formal management, due to the immediate proximity of colleagues when an AE occurs. Coworkers can provide this support even informally, regardless of whether they are not trained in SV response^46^. Empathetic leaders foster an emotionally supportive environment and establish formal and informal support mechanisms, such as counseling services and support groups, to help mitigate the emotional effects of AEs^47^-^50^.

Therefore, it is essential for healthcare institutions to adopt policies and practices that promote a culture of safety, encourage error reporting without fear of punishment, and provide appropriate emotional and psychological support to SVs. Support programs, alongside empathetic and collaborative leadership, are essential to help professionals recover from the trauma of AEs and return to work with safety and confidence. By prioritizing the well-being of healthcare professionals, we not only strengthen a just safety culture but also develop a more humane, resilient, and responsive healthcare system prepared to face the challenges inherent to medical care.
